# Detection of Age-Related Somatic Alterations in Canine Blood Using Next-Generation Sequencing-Based Liquid Biopsy: An Analysis of over 4800 Dogs

**DOI:** 10.3390/vetsci10070455

**Published:** 2023-07-11

**Authors:** Kristina M. Kruglyak, Allison L. O’Kell, Todd A. Cohen, Maggie A. Marshall, Carlos A. Ruiz-Perez, Francesco Marass, John A. Tynan, Susan C. Hicks, Katherine M. Lytle, Ashley Phelps-Dunn, Gina Brandstetter, Chelsea D. Warren, Lauren R. DiMarzio, Michelle C. Rosentel, Lilian K. Wong, Lisa M. McLennan, Jill M. Rafalko, Daniel S. Grosu, Jason Chibuk, Ilya Chorny, Angela L. McCleary-Wheeler, Andi Flory, Dana W. Y. Tsui

**Affiliations:** 1 Information Technology, PetDx, La Jolla, CA 92037, USA; 2 Medical & Clinical Affairs, PetDx, La Jolla, CA 92037, USA; 3 Research Programs, PetDx, La Jolla, CA 92037, USA; 4 Analytical Production, PetDx, La Jolla, CA 92037, USA; 5 Carlson College of Veterinary Medicine, Oregon State University, Corvallis, OR 97331, USA

**Keywords:** clonal hematopoiesis of indeterminate potential (CHIP), age-related clonal hematopoiesis (ARCH), copy number variant (CNV), next-generation sequencing (NGS), somatic, germline, cancer, liquid biopsy, canine, clonal expansion

## Abstract

**Simple Summary:**

In humans there is a biological phenomenon known as CHIP (clonal hematopoiesis of indeterminate potential) in which somatic (acquired) mutations cause blood cells of a certain type to grow disproportionately by making many copies (or “clones”) of themselves. Although most people who are found to have CHIP do not have cancer, they are known to be at higher risk of developing cancer. CHIP has been studied extensively in humans, where it occurs more frequently with increasing age and is thought to be present in 10–20% of people over the of age 70; however, CHIP has not been well-studied in other species. This study provides the first population-level evidence for the potential existence of CHIP-like findings in dogs. Further research is needed to determine the clinical significance of these findings in canine patients.

**Abstract:**

Age-related somatic genomic alterations in hematopoietic cell lines have been well characterized in humans; however, this phenomenon has not been well studied in other species. Next-generation sequencing-based liquid biopsy testing for cancer detection was recently developed for dogs and has been used to study the genomic profiles of blood samples from thousands of canine patients since 2021. In this study, 4870 client-owned dogs with and without a diagnosis or suspicion of cancer underwent liquid biopsy testing by this method. Copy number variants detected exclusively in genomic DNA derived from white blood cells (WBC gDNA-specific CNVs) were observed in 126 dogs (2.6%; 95% CI: 2.2–3.1); these copy number variants were absent from matched plasma cell-free DNA, and from tumor tissue in dogs with concurrent cancer. These findings were more common in older dogs and were persistent in WBC gDNA in over 70% of patients, with little to no change in the amplitude of the signal across longitudinal samples. Many of these alterations were observed at recurrent locations in the genome across subjects; the most common finding was a partial loss on CFA25, typically accompanied by a partial gain on the same chromosome. These early findings suggest that age-related somatic alterations may be present at an appreciable frequency in the general canine population. Further research is needed to determine the clinical significance of these findings.

## 1. Introduction

The phenomenon of somatic genomic alterations in hematopoietic cell lines, commonly referred to as clonal hematopoiesis of indeterminate potential (CHIP) or age-related clonal hematopoiesis (ARCH), has been well characterized in humans and refers to somatic alterations that occur as a result of clonal expansion of the blood system, particularly the white blood cell populations. These alterations tend to be associated with advanced age, often exhibit consistent signal over time, and are frequently found in recurrent locations in the genome [1,2,3,4,5]. Most human patients who have these somatic alterations do not have concurrent cancer. In patients who do have concurrent cancer (specifically those with solid tumors), these alterations are typically not found in the corresponding tumor tissue. In humans, CHIP has been shown to be associated with an increased risk of developing a primary or secondary hematologic malignancy and represents an emerging biomarker for cancer risk prediction. Two population-based studies, each involving more than 10,000 people, have independently shown that the presence of CHIP alterations is associated with a roughly 10-fold higher relative risk of developing hematological cancers when compared to age-matched controls, with an absolute risk of around 0.5% per year [2,3]. For this reason, leading medical centers have established “CHIP clinics” for close clinical monitoring of patients with known CHIP findings [6,7,8,9].

In addition to being a well-documented risk factor for malignancy in humans, CHIP has also been associated with an increased risk for cardiovascular disease (HR 1.9; 95% CI, 1.4–2.7) [10] and may be a risk factor for cerebrovascular events (e.g., stroke) [11,12]. Despite the growing body of literature surrounding CHIP in humans, the population-level prevalence and the clinical correlates of CHIP have not been systematically studied in dogs or other species.

Recently, a blood-based liquid biopsy test using next-generation sequencing (NGS) was developed and clinically deployed for cancer detection in dogs. The clinical validation of this test involved 1100 cancer-diagnosed and presumably cancer-free client-owned dogs [13]. The test has been additionally performed in thousands of dogs since it became commercially available in 2021. This recent ability to test large numbers of dogs using liquid biopsy affords an unprecedented opportunity to study the genomic profiles of a broad population of canine patients. During liquid biopsy testing in dogs, cell-free DNA (cfDNA) is extracted from plasma and genomic DNA (gDNA) is extracted from white blood cells (WBCs) present in the buffy coat; extracted DNA is then subjected to NGS to identify genomic alterations. Cell-free DNA comprises DNA shed from a variety of tissues throughout the body, including tumors (if present). When genomic alterations are identified in cfDNA, this indicates the likely presence of cancer in the body. When genomic alterations are identified in gDNA, this could indicate the presence of a constitutional (germline) abnormality in the patient, including mosaicism; certain hematologic malignancies (when corresponding CNVs are also identified in cfDNA); or age-related somatic alterations (e.g., CHIP).

Most studies in humans have focused on characterizing single nucleotide variants (SNVs) in particular genes associated with CHIP, but recent reports have shown that CHIP-associated copy number variants (CNVs) are also an important risk factor for the development of leukemia and cardiovascular disease [4,5,10]. NGS-based liquid biopsy offers an opportunity to study if similar alterations are seen in dogs and whether they may be useful as biomarkers to predict the risk for cancer or other diseases.

The current study aimed to evaluate the population-level frequency of age-related somatic copy number alterations in the WBC gDNA of dogs and to characterize the type of alterations observed in these patients. Additional studies are currently underway to evaluate the clinical significance of these findings; if successful, these studies could pave the way for broad adoption of CHIP-based testing and monitoring approaches in veterinary medicine that are similar to those already employed for human patients.

## 2. Materials and Methods

A total of 4870 client-owned dogs were evaluated as part of this study. Of this total, whole blood samples from 3595 dogs with and without a clinical suspicion of cancer were submitted by the dog’s veterinarian for commercial liquid biopsy testing at the PetDx laboratory in La Jolla, CA (“clinical cohort”); whole blood samples from 1275 dogs with and without a diagnosis of cancer were obtained as part of a larger research collection to support the clinical validation of the NGS-based liquid biopsy test (“research cohort”) [13]. All research subjects were enrolled under protocols that received Institutional Animal Care and Use Committee (IACUC) or site-specific ethics approval [13]. All methods were performed in accordance with the relevant guidelines and regulations and follow the recommendations in the ARRIVE guidelines. All research subjects were client-owned, and written informed consent was obtained from all owners. In a subset of cancer-diagnosed patients from the research cohort, matched tumor tissue samples were also available for analysis. Additionally, a subset of patients across both the clinical and the research cohorts submitted whole blood samples at multiple timepoints, allowing for longitudinal monitoring of genomic alterations.

Whole blood samples were collected from each patient; cfDNA was extracted from plasma, and gDNA was extracted from WBCs present in the buffy coat; in the subset of patients with matched tumor tissue available, DNA was also extracted from tissue [13,14]. All extracted DNA specimens were subjected to proprietary library preparation and NGS as previously described [13]. Sequencing data were analyzed using an internally developed bioinformatics pipeline to determine the presence of genomic alterations.

**Figure 1:**
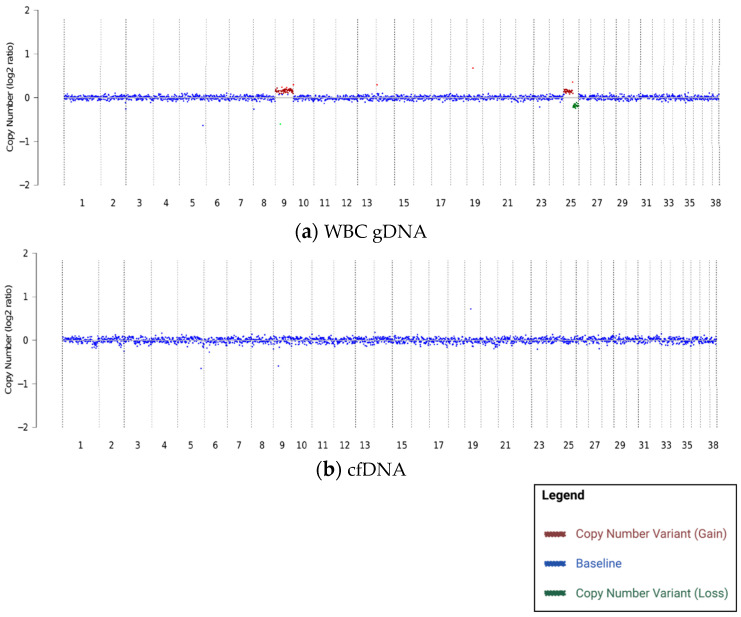
Example of a patient with WBC gDNA-specific CNVs where (**a**) CNVs were identified in WBC gDNA (**b**) but not in cfDNA in an 11-year-old neutered male mixed-breed dog with no clinical evidence of cancer.

This study focused on a large cohort of canine patients in which specific CNVs were identified in WBC gDNA but were absent in matched cfDNA samples. These findings will be referred to as “WBC gDNA-specific CNVs”, and one example is shown in Figure 1. It should be noted that a subset of patients with WBC gDNA-specific CNVs also had concurrent CNVs identified in cfDNA (and/or tissue, when available) but those CNVs were different from the CNVs identified in WBC gDNA.

A subset of dogs with WBC gDNA-specific CNVs had clinical evaluations performed to determine the presence of cancer. Though the components of each evaluation varied, they typically comprised a thorough physical examination, laboratory workup (CBC, chemistry panel, and urinalysis), imaging (thoracic radiographs and/or abdominal ultrasound), and tissue sampling (via fine needle aspiration or biopsy) of observed masses, lesions, and/or enlarged lymph nodes.

When the clinical evaluation determined that cancer was present, a definitive or presumptive cancer diagnosis was assigned. “Definitive” diagnoses were those in which cancer was confirmed via tissue-based testing (cytology or histopathology). “Presumptive” diagnoses were based on imaging, direct visualization/exam, or a strong suspicion from cytology or histopathology.

To compare the age profiles of adult dogs with and without WBC gDNA-specific CNVs, a “normalized age” was calculated relative to expected lifespan, where expected lifespan was calculated as a function of weight according to the equation: lifespan = 13.987 − 0.035 × weight [15]. *p*-values were calculated using a two-sided *t*-test. Analyses were performed using R (version 4.0.5).

## 3. Results

### 3.1. Population Characteristics

The study population of 4870 client-owned dogs comprised 3595 dogs from the clinical cohort and 1275 dogs from the research cohort (Table 1).

In the clinical cohort, cancer status was unknown at the time of sample submission. Samples for 962 dogs were submitted due to a clinical suspicion of cancer (liquid biopsy was used as an aid in diagnosis); samples for 2399 dogs were submitted with no reported suspicion of cancer (liquid biopsy was used as a screening test); and samples for 234 dogs were submitted without documentation regarding cancer suspicion. The average age of dogs in the clinical cohort was 9.09 years (SD = 3.06; *n* = 3595) and the average weight was 25.80 kg (SD = 13.48; *n* = 3144).

In the research cohort, 569 dogs had a definitive diagnosis of cancer and 706 dogs were presumably cancer-free. The average age of dogs in the research cohort was 7.17 years (SD = 3.68; *n* = 1275) and the average weight was 28.28 kg (SD = 12.04; *n* = 1275). A wide range of purebred and mixed-breed dogs were represented in both cohorts.

### 3.2. Dogs with WBC gDNA-Specific CNVs and Their Associated Clinical Status

WBC gDNA-specific CNVs were identified in 164 of the 4870 (3.4%; 95% CI: 2.9–3.9) dogs in the overall study population: 129 from the clinical cohort and 35 from the research cohort. For 126 of these dogs (107 from the clinical cohort and 19 from the research cohort), CNVs were identified only in the WBC gDNA, and no CNVs of any kind were observed in plasma-derived cfDNA. (Table 2).

Clinical cancer evaluations were performed in 42 of the 126 dogs with WBC gDNA-specific CNVs: 28 from the clinical cohort and 14 from the research cohort.

Of the 28 patients in the clinical cohort, 18 had samples submitted with no suspicion of cancer at the time of blood draw (i.e., the test was used for cancer screening). Within this group, 78% (14/18; 95% CI: 51.9–92.6) had no evidence of cancer following clinical evaluation, while 22% (4/18; 95% CI: 7.4–48.1) received a definitive or presumptive cancer diagnosis. The four cancer diagnoses in the screening patients included: splenic stromal sarcoma (definitive, diagnosed via histopathology), a splenic mass that was suspected to be cancer on imaging but did not have tissue testing for confirmation (presumptive), a hepatic mass (presumptive), and an adrenal gland tumor (presumptive). 

The remaining 10 out of 28 patients in the clinical cohort had samples submitted for testing due to suspicion of cancer (i.e., the test was used as an aid in diagnosis). Within this group, 50% (5/10; 95% CI: 20.1–79.9) had no evidence of cancer following clinical evaluation, while 50% (5/10; 95% CI: 20.1–79.9) received a definitive or presumptive cancer diagnosis. The 5 cancer diagnoses in the aid-in-diagnosis patients included: histiocytic sarcoma (definitive), a mixed germ cell-sex cord stromal tumor (definitive), two bone tumors (both presumptive), and an adrenal gland tumor (presumptive).

All 14 dogs whose samples were submitted as part of the research collection had a definitive diagnosis of cancer at the time of blood collection. The cancer diagnoses in these dogs were as follows: lymphoma/acute lymphoid leukemia (4 cases), osteosarcoma (3 cases), mast cell tumor (2 cases), hemangiosarcoma, anal sac adenocarcinoma, chronic lymphoid leukemia, soft tissue sarcoma, and transitional cell carcinoma. 

There were 38 dogs with CNVs identified in plasma-derived cfDNA in addition to WBC gDNA-specific CNVs (22 from the clinical cohort and 16 from the research cohort); 26 of these dogs (11 from the clinical cohort and 15 from the research cohort) had clinical cancer evaluations performed. Among those in the research cohort, all were among the 569 dogs who were enrolled with a definitive diagnosis of cancer. The cancer confirmation rate for these 26 dogs was 100% (26/26; 95% CI: 84.0–100), demonstrating that observations of somatic CNVs in the plasma cfDNA are highly predictive of concurrent cancer.

### 3.3. WBC gDNA-Specific CNVs Not Present in Matched Tumor Tissue When Cancer Is Concurrently Present

Tumor tissue samples (from the same collection timepoint as the blood samples with WBC gDNA-specific findings) were available for testing by NGS from 11 of the 35 cancer-diagnosed dogs in the research cohort. The CNVs observed in gDNA were not present in the matched tumor tissue in 91% of cases (10/11). In one case, the same CNVs observed in WBC gDNA were present in matched tissue (from a fine needle aspirate of the left mandibular lymph node); this subject had a definitive diagnosis of T-zone lymphoma (stage IIa).

**Figure 2:**
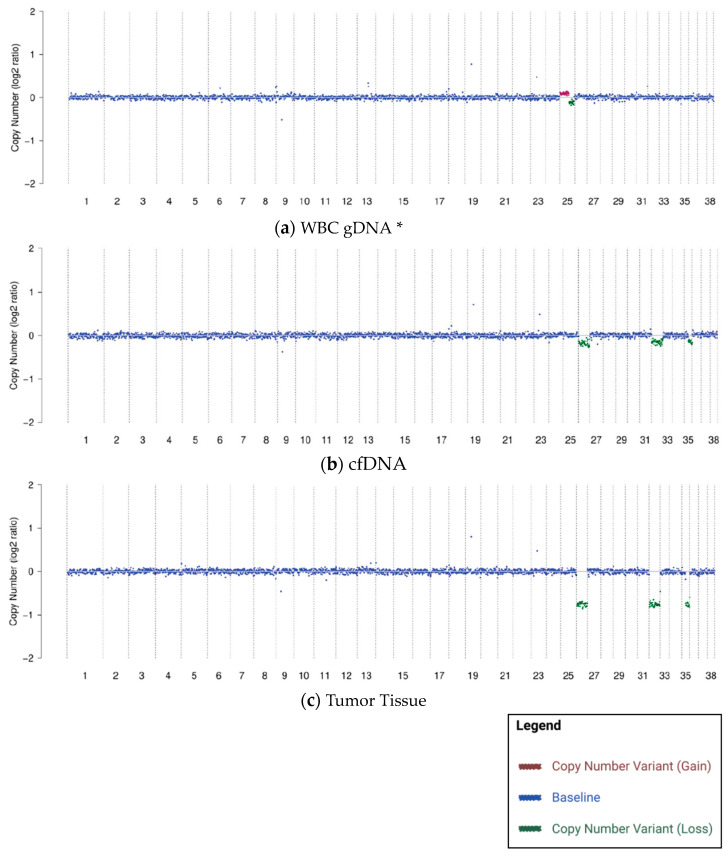
CNVs identified in WBC gDNA (**a**) that are different from CNVs in cfDNA (**b**) and from CNVs in tumor tissue (**c**) in a 10-year-old neutered male mixed-breed dog with hepatocellular carcinoma. * Color added to sequencing traces for visualization of CNVs.

Tumor tissue samples (from the same collection timepoint as the blood samples with cfDNA-specific CNV findings) were available for testing by NGS from 105 cancer-diagnosed dogs in the research cohort. In contrast to WBC gDNA-specific CNVs, the CNVs observed in cfDNA did match the CNVs found in tumor tissue in the vast majority of cases (92%; 97/105). Figure 2 shows the matched CNV profiles from one subject with CNVs present in tissue, cfDNA, and gDNA. The WBC gDNA-specific CNVs on CFA25 (canine chromosome 25; Figure 2a) are not visible in the tumor tissue (Figure 2c); however, the cfDNA-specific CNVs on CFA26, CFA32, and CFA35 (Figure 2b) are clearly visible in the tumor tissue.

These results suggest that WBC gDNA-specific CNVs, when present, are typically coincidental (but not biologically related) to the patient’s cancer, while cfDNA-specific CNVs are typically derived from the patient’s cancer.

### 3.4. WBC gDNA-Specific CNVs Persistent across Longitudinal Samples

**Figure 3:**
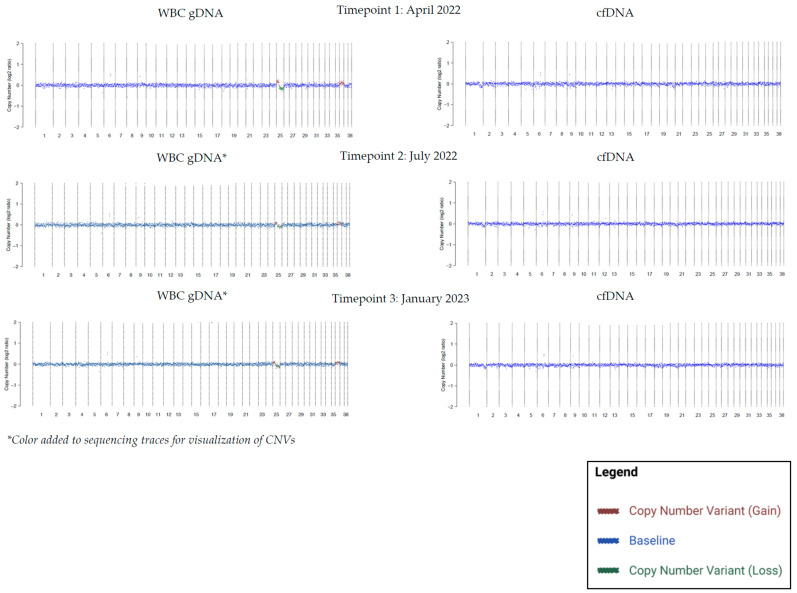
Longitudinal blood samples from a 13-year-old spayed female mixed-breed dog with no history of cancer and no suspicion of cancer showing persistence of a chromosome 25 (CFA25) gain/loss and a chromosome 36 (CFA36) gain in WBC gDNA with little to no change in the amplitude of the signal, and absence of this finding in cfDNA over a 9-month period.

Longitudinal blood samples were available for a total of 76 patients with WBC gDNA-specific findings. Seventy-one percent of patients (54/76; 95% CI: 60.0–80.0) showed persistence of the gDNA CNVs over time. Figure 3 shows CNV profiles from one of these patients, with persistent WBC gDNA-specific CNVs in CFA25 and CFA36 in three consecutive timepoints spanning 9 months. In the remaining 29% (22/76; 95% CI: 19.4–40.7) of cases, the gDNA CNVs were not persistent across all subsequent timepoints.

### 3.5. WBC gDNA-Specific CNVs at Recurrent Locations in the Genome

Several WBC gDNA-specific CNVs were recurrent across patients; the most common CNV was a partial loss of chromosome 25, typically accompanied by a partial gain on the same chromosome (Table 3; Figure 1a and Figure 2a). This pattern is consistent with human literature, where CHIP has been reported at recurrent locations in the genome [4,5,16].

### 3.6. WBC gDNA-Specific CNVs More Frequently Observed in Older Dogs

**Figure 4:**
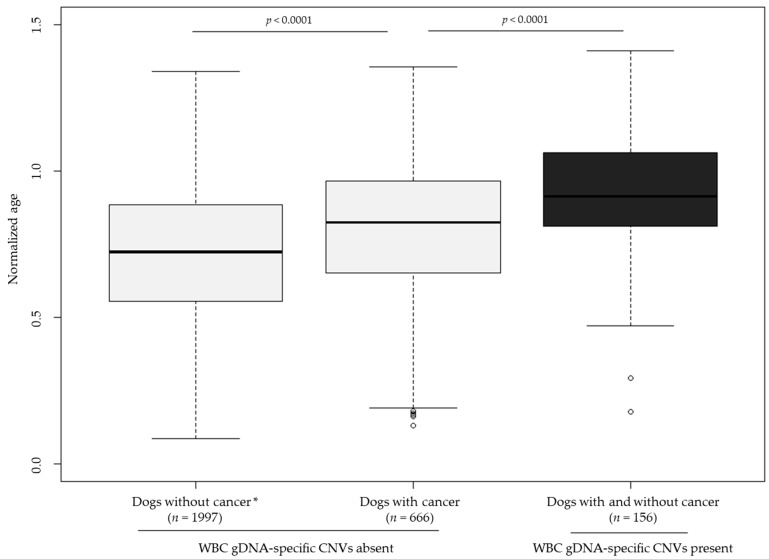
Normalized age distributions for dogs with and without cancer and absence of WBC gDNA-specific CNVs, compared to dogs with and without cancer and presence of WBC gDNA-specific CNVs. Analysis focused on dogs for which normalized age could be calculated (i.e., adult dogs with both age and weight reported). * Dogs without cancer comprised clinical cohort samples that were submitted with no suspicion of cancer (i.e., for screening) and that had no evidence of cancer from liquid biopsy testing (i.e., no somatic genomic alterations detected in cfDNA). Samples from the clinical cohort submitted due to a suspicion of cancer (i.e., as an aid in diagnosis) and samples with evidence of cancer from liquid biopsy testing were excluded.

The study cohort was analyzed to evaluate the normalized age of patients with and without WBC gDNA-specific CNVs, and their cancer status. Among patients without WBC gDNA-specific CNVs, dogs with a diagnosis of cancer (definitive or presumptive) were significantly older than dogs who submitted samples as part of the clinical cohort for the purpose of screening and had no evidence of cancer (*p* < 0.0001); in addition, patients with WBC gDNA-specific findings (whether or not they had cancer) were significantly older than the cancer-diagnosed dogs without WBC gDNA-specific CNVs (*p* < 0.0001). (Figure 4) No significant difference was observed in the age distribution of patients with WBC gDNA-specific CNVs as a function of cancer status (*p* = 0.5864).

## 4. Discussion

The identification of recurrent CNVs in the WBC gDNA of older dogs, which persist with little to no change in amplitude over time and are absent from cfDNA (and from tumor tissue, in dogs with concurrent cancer), provides early evidence for the existence of age-related somatic alterations in dogs that resemble the phenomenon of CHIP previously described in humans.

Most of the published research on CHIP in humans involves analysis of SNVs [2,3]; recent studies have also reported CHIP-associated CNVs that are likewise age-related and are associated with a higher risk of the development of leukemia in individuals with or without other existing cancer [4,5,16]. Additionally, these studies have shown that the co-occurrence of CHIP-associated CNVs and SNVs was associated with a higher cumulative incidence of leukemia than the exclusive occurrence of either type of genomic alteration. A recent study using blood samples from cancer-diagnosed dogs found that a small percentage (4.3%; 4/93) of subjects had genomic alterations (SNVs) in genes that have been associated with CHIP in human patients; however, no tumor tissue samples were available to confirm that these alterations did not in fact originate from the cancer itself, and no comparisons were made between WBC-derived gDNA and plasma-derived cfDNA [17].

In the current study, analysis was focused on WBC gDNA-specific CNVs in a large cohort of canine patients. Additional studies are underway at PetDx to evaluate the prevalence and distribution of WBC gDNA-specific SNVs in large populations of dogs, as well as the interplay between multiple types of CHIP-associated genomic alterations and clinical outcomes.

The clinical significance of age-related somatic alterations in dogs that do not currently have a diagnosis of cancer is uncertain at present and requires further research. The current study found that 22% (4/18; 95% CI: 7.4–48.1) of dogs with WBC gDNA-specific findings (in the absence of cfDNA findings) but no initial suspicion of cancer (i.e., screening patients) received a definitive or presumptive diagnosis of cancer following a clinical evaluation. It is unclear how this number compares to the actual prevalence of cancer in an age-matched canine cancer screening population. It will be important to prospectively follow cancer-free dogs with WBC gDNA-specific findings to determine whether they are at higher risk of developing cancer in the future compared to their age-matched counterparts. Long-term monitoring studies are ongoing for patients with WBC gDNA-specific findings identified during clinical testing at PetDx. 

CHIP in humans is also associated with an increased risk of cardiovascular and potentially cerebrovascular disease [10,11,12]. It is currently unknown whether the presence of WBC gDNA-specific findings confers a similar risk for these or other conditions in canine patients. Though the current study was not designed to address this question, longitudinal monitoring of dogs with these genomic findings may help to provide such information.

In addition to the current lack of longitudinal outcome data from patients with WBC gDNA-specific findings, this study faces other limitations.

CNVs identified in WBC gDNA have not yet been confirmed to originate from a single hematopoietic cell type, which would substantiate their clonal nature; research is ongoing to elucidate the specific biological source(s) of these findings.

Additionally, normalized age calculations were based exclusively on weight for all dogs without consideration of owner-reported breed, which may result in overestimation or underestimation in the case of specific breeds.

Lastly, not all dogs had complete medical records and/or clinical evaluations to determine the presence of cancer.

## 5. Conclusions

The large-scale NGS-based genomic profiling of dogs with and without cancer has uncovered the first potential evidence of age-related somatic copy number alterations in dogs at the population level and is likely to reveal additional novel findings as the use of this type of testing expands. It is not currently known if CHIP-like genomic alterations confer a similar risk of cancer (particularly hematological malignancies) or other conditions (such as cardiovascular disease) in dogs as they do in humans. Continued research, including long-term clinical monitoring of affected canine patients, will be required for proper clinical interpretation of such findings in the context of liquid biopsy testing in dogs. Such studies have the potential to validate CHIP as an independent risk factor for cancer in dogs and pave the way for the development and clinical adoption of NGS-based CHIP testing and monitoring strategies similar to those now being used in people.

## Figures and Tables

**Table 1:** Demographics of dogs in the clinical and research cohorts.

	Clinical Cohort (*n* = 3595) N (%)	Research Cohort (*n* = 1275) N (%)
**Cancer status**		
Cancer suspected	962 (26.8%)	-
Cancer not suspected	2399 (66.7%)	-
Cancer suspicion not provided	234 (6.5%)	-
Cancer-diagnosed	-	569 (44.6%)
Presumably cancer-free	-	706 (55.4%)
**Sex**		
Male	1797 (50.0%)	665 (52.2%)
*Neutered*	1581	572
*Intact*	204	92
*Not provided*	12	1
Female	1750 (48.7%)	610 (47.8%)
*Spayed*	1608	553
*Intact*	96	56
*Not provided*	46	1
Sex not provided	48 (1.3%)	0 (0%)
**Age (years)**		
Mean (SD)	9.1 (3.1)	7.2 (3.7)
**Weight (kg)**		
Mean (SD)	25.8 (13.5) *	28.3 (12.0)
**Breed classification**		
Purebred	2198 (61.1%)	630 (49.4%)
Mixed-breed or unknown	1397 (38.9%)	645 (50.6%)

* Weight was available for 3144 dogs in the clinical cohort.

**Table 2:** Disposition of dogs in the clinical and research cohorts based on the presence or absence of gDNA-specific CNVs and concurrent CNVs in plasma-derived cfDNA.

	Clinical Cohort (*n* = 3595) N; % [95% CI]	Research Cohort * (*n* = 1275) N; % [95% CI]
**gDNA-specific CNVs present without concurrent cfDNA CNVs**	**107; 3.0 [2.5, 3.6]**	**19; 1.5 [0.9, 2.4]**
Cancer evaluation performed	28	14
Cancer	9	14
No cancer	19	0
No additional cancer evaluation following	79	5
liquid biopsy		
**gDNA-specific CNVs present with concurrent cfDNA CNVs**	**22; 0.6 [0.4, 0.9]**	**16; 1.3 [0.7, 2.1]**
Cancer evaluation performed	11	15
Cancer	11	15
No cancer	0	0
No additional cancer evaluation following	11	1
liquid biopsy		
**gDNA-specific CNVs absent**	**3466; 96.4 [95.8, 97.0]**	**1240; 97.3 [96.2, 98.0]**
Cancer evaluation performed	422	549
Cancer	124	542
No cancer	298	7
No additional cancer evaluation following	3044	691
liquid biopsy		

* The term “cancer evaluation” in the context of this study refers to a thorough workup performed when cancer is suspected (because of liquid biopsy results and/or because of the patient’s clinical presentation); the components of this workup are described in Methods. It should be noted that all presumably cancer-free dogs in the research cohort had a clinical history and physical exam at the time of study enrollment.

**Table 3:** Recurrent copy number variants identified in WBC gDNA and their frequency in the population of dogs with WBC gDNA-specific CNVs.

Chromosome	Gain/Loss	Number of Dogs with This Finding *	% of Dogs with WBC gDNA-Specific CNVs with This Finding * % [95% CI]
25	Loss	46	28.1 [21.7, 35.4]
25	Gain	39	23.8 [17.6, 31.2]
16	Loss	22	13.4 [8.8, 19.8]
9	Gain	21	12.8 [8.3, 19.1]
14	Gain	18	11.0 [6.8, 17.0]
13	Gain	14	8.5 [4.9, 14.2]
8	Loss	13	7.9 [4.5, 13.5]
36	Gain	10	6.1 [3.1, 11.2]

* Of the 164 dogs with WBC gDNA-specific CNV(s); Findings were not mutually exclusive. Some dogs had multiple concurrent WBC gDNA findings, either within a single chromosome (e.g., a partial gain and a partial loss of chromosome 25) or affecting multiple chromosomes (e.g., gains on both chromosomes 14 and 25).

## Data Availability

All relevant data are contained within this article.
